# Voluntary Hospital Employees and the National Insurance Act

**Published:** 1912-03-02

**Authors:** 


### VOLUNTARY HOSPITAL EMPLOYEES AND THE NATIONAL INSURANCE ACT.

### Practical Considerations of Urgent Importance.

The Board of Governors of the Leicester Infirmary at their meeting on the 21st ultimo had before them a memorandum prepared by the house-governor and secretary, Mr. Harry Johnson, which tersely summarises those portions of the Act which relate to approved societies, the meaning of voluntary and deposit contributors, the Government's contributions, the benefits offered, with the defined exceptions and reductions, and reserve funds.

### Persons to be Insured.

Mr. Johnson then sets forth under this heading the employees of hospitals who will be affected by the Act as follows:—

Nursing staffs (but see Section 51), domestic staffs, dispensers, clerks, porters and attendants, carpenters and joiners, painters, etc.

Nursing Staff: Hitherto regarded as a matter of custom in hospitals to provide medical attendance. Medical examination on entrance for training, picked lives. Sickness comparatively light. In the interests of the institutions that present methods be continued. Probably few hospitals will elect for nurses to go on sick fund, except in cases of severe illness, during their training or service.

Domestic Staff: Treatment customarily given in institution.

Male Servants: Will probably receive treatment at home in minor illnesses and in severe illnesses in institution.

### Figures Taken into Consideration.

Under this title Mr. Johnson proceeds to give the total number of beds in London and English provincial hospitals, large and small, computed for purposes of present calculations at 25,000. He states that the figures he gives relating to hospital staffs are taken from Burdett's "Hospitals and Charities," 1911, and sets them forth in the following table:—

| | |
|---|---|
| Average number of nurses employed per bed, 3, taken for purposes of these figures as 5, hence total number of nurses employed, 525,000 = | 5,000 |
| Estimate of number of porters, mechanics, employed in English hospitals ... ... ... | 2,500 |
| Clerical staff, say ... ... ... ... ... ... | 750 |
| Dispensing staff, say ... ... ... ... ... | 350 |
| Domestic staff (including wardmaids), say ... | 2,500 |
| Laundry hands, say ... ... ... ... ... ... | 1,000 |
| | 12,100 |

Deduct proportion of above who may be already insured in Friendly Societies, etc., say one-fourth = 3,000, leaving 9,000—amply sufficient to form an approved society.

N.B.—No account is taken of employees of convalescent homes or orphanages, homes for incurables, nursing associations, and the like, which might possibly be grouped with hospitals for the purposes of the Act.

The figures given are believed to be under-estimated.

It must be observed in regard to these figures that they are estimates merely, for at the present time there are no complete records of the numbers of persons employed in hospitals and kindred institutions in the various capacities set forth in Mr. Johnson's table. We may point out however that, so far as the estimated number of nurses is concerned, namely, 5,000, the report from the House of Commons Select Committee of August 1905 on the Registration of Nurses contains appendices in which Sir Henry Burdett supplies a return giving a summary including all nursing officials, nurses, private nurses and probationers employed in 499 hospitals and Poor Law infirmaries of fifty beds and upwards, and in certain nursing institutions managed by a committee, and Government Departments in the United Kingdom, in January 1905, which amounted to 19,789, viz., 11,038 nurses including matrons and all officers, 7,062 probationers, and 1,689 private nurses.

### Trained Nurses and National Insurance.

The time has arrived, no doubt, when every hospital committee should consider what course it will be best to recommend the various sections of their staff to take in order to secure for themselves the fullest advantages obtainable under the National Insurance Act. These questions have been engaging the earnest attention of the Council of the Royal National Pension Fund for Nurses, which has been in close communication with Government authorities in regard to these matters especially, so far as they might affect the nurses, from the first introduction of the Insurance Bill. One result of the Pension Fund action has been to secure in the Act authority whereby this Fund can establish a separate section, which is to become an "approved society" in order to carry out the requirements of the National Insurance Act. This separate section will be known as the Nurses' National Insurance Society; it will not be open to men, whether nurses or otherwise, but will be open to all other nurses, whether members of the Pension Fund or not. A strong representative advisory committee of matrons and nurses will assist in framing the rules of this society. Directly the necessary information is received from the Insurance Commissioners, these rules will be completed and issued. It will thus be seen that, under the highest financial guarantees, trained nurses of all grades throughout the United Kingdom, and indeed the Empire, will be able to obtain the utmost advantages which the National Insurance Act has to offer them by becoming members of the Nurses' National Insurance Society.

### Hospital Employees other than Nurses.

When we come to consider the position of hospital employees other than nurses, who Mr. Johnson calculates will number at least 7,000, the problem presented is one not easy of solution. It may be well to state here, what is not known generally, that the constitution of the Royal National Pension Fund for Nurses provides amongst the objects of the Fund that it may include all descriptions of officials of hospitals and other kindred institutions, and all other institutions whose objects embrace, directly or indirectly, the relief or assistance of the sick, the injured, or the distressed. It is therefore clear that the Council has the power, should there be sufficient demand for it, to include in a separate section male employees of hospitals, and all other employees too, should such a course be found practicable, seeing that the Insurance Commissioners have now definitely stated that it will be legally possible to form two separate sections, one for men and one for women. Were there to be one society to include both men and women employees—that is to say, one with two branches in the same section, one for men and one for women —surpluses and deficiencies could not be kept separate. Now it is essential that a separate section shall be organised for nurses only, seeing that otherwise they might suffer very considerably in benefit, as the risks which they undergo differ materially from those of ordinary hospital employees. It follows that if all hospital employees other than nurses are to be provided for, through the Pension Fund or otherwise, there must be set up a separate society for them, distinct altogether from the nurses' section.

### Provision for Employees other than Nurses.

At the meeting on February 21 of the Board of the Leicester Infirmary, on the motion of Sir Edward Wood, chairman of the board, seconded by Mr. T. Fielding Johnson, chairman of the house committee, it was resolved:

"That in view of the information submitted to the Board of Governors of the Leicester Infirmary, a suggestion be made to the British Hospitals Association to appoint a committee to consider the advisability of the English hospitals combining for the purpose of forming an Approved Society under Section 23 of the National Insurance Bill, and in the event of the British Hospitals Association not being able to adopt this suggestion, the English hospitals be circularised with a view to an early meeting being called to discuss the advisability of forming an Approved Society under the Act, and a competent authority on the Act be invited to address the meeting."

This resolution will be considered, we understand, at a meeting of the British Hospitals Association on March 1, after we go to press. It is a grave and by no means a simple matter for hospital committees to determine what will be the wisest and best course to adopt in regard to the policy included in the Leicester resolution. It is manifest that in the first instance hospital managers, or many of them, may determine to take the prudent course of conferring with the Council of the Royal National Pension Fund for Nurses, so that a decision may be arrived at as to whether or not this Council will be prepared to co-operate with the hospitals in the formation of an approved society for men employees in hospitals and kindred institutions, or, in the alternative, for men and women employees other than trained nurses? It will certainly be most desirable to unite all the hospitals, if such a course should be found practicable. We foresee, however, that whether this latter society is organised through the Pension Fund or independently, the difficulties to be overcome are neither few nor unimportant. We propose to deal with these questions more fully next week.

### Points to be First Determined.

So far as hospital employees are concerned, the first great point to determine will be whether each such employee on leaving the service of the hospital must resign his or her membership in this society. Unless this is made compulsory, it is highly probable that the number of members outside hospital workers may speedily become much greater than the actual number of hospital-workers members, with the result that the outside element would control and manage the society. This is important, because the Leicester committee seem to be of opinion that it cannot be in the best interests of the hospitals themselves to have some nurses in one and some in another society, domestic servants in another, laundresses perhaps in another, and workmen scattered amongst the various friendly societies. No hospital board could take much practical interest in their staff under such circumstances, but it is felt that they could and would take a deep personal interest if they were sure that they could share an interest in a central fund.

### No Hospital Employee can be Bound.

Another point in this connection is that no hospital committee will, we presume, attempt, nor could they bind any employee legally, we believe, that they should join this or that society and no other. Once again, in the matter of women other than nurses employed in hospitals, several hospital authorities hold that it would be inadvisable to include cooks, housemaids, ward-maids, laundry-maids, and others, for it is found by experience that, many of them engage themselves to hospitals in order to have their health looked after, and especially their teeth, and that they are apt to move on to other employment when their health is perfectly restored. To mention such points is but to indicate the care and foresight which must be exercised if the wisest decision and most perfect arrangements are to be made to adapt the National Insurance Act to the special requirements of every section of hospital employees and of all the hospitals and kindred institutions too.

### Co-operation and Information.

It would be a material help to the solution of these various points if the managers of the voluntary hospitals would send us their views and opinions. We are making all arrangements to open a National Insurance Section, which will be conducted by an expert with actuarial assistance, so that the whole of the voluntary hospitals and kindred institutions, and all the employees may readily obtain free of cost all the help and information they may need in the matter. We hope in this way to make The Hospital the friend of all who are interested in National Insurance.

